# Developing a Data Dashboard Framework for Population Health Surveillance: Widening Access to Clinical Trial Findings

**DOI:** 10.2196/11342

**Published:** 2019-04-04

**Authors:** David Concannon, Kobus Herbst, Ed Manley

**Affiliations:** 1 Centre for Advanced Spatial Analysis University College London London United Kingdom; 2 Africa Health Research Institute Durban South Africa

**Keywords:** data visualization, data dashboards, health and demographic surveillance, sub-Saharan Africa, treatment as prevention, clinical trials, demographics, real-time, data literacy

## Abstract

**Background:**

Population surveillance sites generate many datasets relevant to disease surveillance. However, there is a risk that these data are underutilized because of the volumes of data gathered and the lack of means to quickly disseminate analysis. Data visualization offers a means to quickly disseminate, understand, and interpret datasets, facilitating evidence-driven decision making through increased access to information.

**Objectives:**

This paper describes the development and evaluation of a framework for data dashboard design, to visualize datasets produced at a demographic health surveillance site. The aim of this research was to produce a comprehensive, reusable, and scalable dashboard design framework to fit the unique requirements of the context.

**Methods:**

The framework was developed and implemented at a demographic surveillance platform at the Africa Health Research Institute, in KwaZulu-Natal, South Africa. This context represents an exemplar implementation for the use of data dashboards within a population health-monitoring setting. Before the full launch, an evaluation study was undertaken to assess the effectiveness of the dashboard framework as a data communication and decision-making tool. The evaluation included a quantitative task evaluation to assess usability and a qualitative questionnaire exploring the attitudes to the use of dashboards.

**Results:**

The evaluation participants were drawn from a diverse group of users working at the site (n=20), comprising of community members, nurses, scientific and operational staff. Evaluation demonstrated high usability for the dashboard across user groups, with scientific and operational staff having minimal issues in completing tasks. There were notable differences in the efficiency of task completion among user groups, indicating varying familiarity with data visualization. The majority of users felt that the dashboards provided a clear understanding of the datasets presented and had a positive attitude to their increased use.

**Conclusions:**

Overall, this exploratory study indicates the viability of the data dashboard framework in communicating data trends within population surveillance setting. The usability differences among the user groups discovered during the evaluation demonstrate the need for the user-led design of dashboards in this context, addressing heterogeneous computer and visualization literacy present among the diverse potential users present in such settings. The questionnaire highlighted the enthusiasm for increased access to datasets from all stakeholders highlighting the potential of dashboards in this context.

## Introduction

Demographic surveillance, the process of monitoring births, deaths, causes of deaths, and migration in a population over time is 1 of the cornerstones of public health research [1,2]. The availability of detailed data on these population statistics is essential to the planning, implementation, and evaluation of any public health intervention [3]. The lack of vital information presents significant challenges for the use of an evidence-based decision making process for public health interventions [4]. Health and Demographic Surveillance Systems (HDSSs) comprise the continuous monitoring of demographic and health characteristics of a population living in a well-defined geographic area [5]. The goal of an HDSS is to generate high-quality longitudinal datasets to capture the demographic and health changes of the set population [6]. The production of large and complex datasets required to monitor disease burden poses many challenges on the public health community to explore, analyze, and extract valuable information to make timely decisions [7]. The World Health Organization has cited the accumulation of unanalyzed data as a challenge facing HDSS sites [3]. AbouZahr et al [8] suggest that because of the vast quantities of data produced by health information systems, the information overload at higher levels is such that datasets are, in practice, seldom used effectively for decision making. For decision makers, it is essential to rapidly extract relevant information from the flood of data [9]. Without the timely analysis of these datasets, there is a decrease in their value as a tool to monitor disease trends [10]. The intelligent implementation of information visualization has been employed in public health and could offer a means to increase the use of datasets at HDSSs.

Information visualization offers a means to disseminate information promptly and increase its visibility among stakeholders. Card et al [11] defined data visualization as the use of visuals to *amplify cognition* to aid task completion. The use of visualization acts as an intermediate step in converting data into information. Visualization tools exploit human visual and spatial skills by using interactive visual representations of data. Visualization can aid decision making, helping the user build accurate mental models that can leverage cognitive skills [12-15]. Research in cognitive capacity demonstrates that humans can process more information presented graphically than in text [16,17]. Keim [9] states that the key benefits provided by visuals are that they act as a frame or a temporary storage area for human cognitive processes.

La Valle [18] highlighted visualization as a useful tool for gaining insight into large and complex datasets. Longitudinal datasets produced by HDSSs are typically multivariate, complex, with varying levels of granularity [17], and thus may represent suitable datasets for the use of visualization tools. Datasets produced at HDSS sites are typically accessible only in formats that do not allow for the rapid extraction and analysis of salient information. The role that surveillance data produced at HDSSs can play in planning new health interventions can be diminished if the data are not communicated in a timely and understandable manner [19]. Given the multiple data sources collected in HDSSs and the variety of potential users, a dashboard is an excellent vehicle for visualization in this context. Few [20] describes dashboards as a consolidated visual display of pertinent information, arranged so the entire operation of an observed system can be monitored and understood at a glance. Wexler et al [21] give a broad definition as “a visual display of data used to monitor conditions and/or facilitate understanding”.

Dashboards typically comprise a combination of different visualization methods; the commonality is that they draw from multiple data sources to facilitate timely understanding [22]. Carroll’s [23] review of the impact of visualization tools on epidemiology highlighted the richness of the information offered by visualization for communication, and decision making is counterbalanced by difficulties in displaying and interpreting these datasets.

Although there is little evidence in the literature of dashboard implementations at HDSS sites, there is a variety of examples relating more generally to public health monitoring. Cheng [19] developed a Web-based interface to share Hong Kong’s flu surveillance data. The design is based on public health websites and dashboard design guidelines. Cheng emphasizes the need to develop a framework with standard datasets to include multiple sources and to visualize the various diseases. Sopan et al [24] built the community health map to share public health data among a variety of different users, for example, policy makers, journalists, and the public. The dashboard focused on the ability to compare different datasets and indicators, including the spatial visualization of data. The results from the pilot suggested a comprehensive visualization interface could lead to better informed decision making. Kostkova et al [25] developed the *medi+board* to coalesce multiple disease surveillance datasets promptly. This study emphasized the role of modularly in design to allow the timely deployment of dashboards. The lessons from these examples have been incorporated into the design of our dashboard framework.

This paper presents the development and evaluation of a design framework for the implementation of a data dashboard within an HDSS context. The paper proceeds by outlining the structure and design rationale behind the framework, with a focus on its implementation within a specific HDSS context. Following this, an evaluation is described, which outlines the implementation of its use within a specific trial context, revealing promising findings concerning task completion, usability, and sentiment. The contributions of this study are guidelines for designing visualization systems for HDSS sites and identifying their information needs and the development of a framework to reflect the needs of the diverse users. The research identifies the challenges of different users in dashboard design and offers suggestions on how to account for this in future systems.

## Methods

### Stages of Data Dashboard Development

The development of a data exploration dashboard framework requires a range of implementation decisions. These factors involve elements of data design, user interaction, and the study setting of the dashboard design, all of which contribute to the potential success of the dashboard. The outline framework described below relates to the design and implementation of a dashboard within a population health–monitoring setting. In developing a dashboard in this context, there are 5 core considerations:

1. Study setting (description of the development context of the framework) informs how interaction with the dashboard takes place, informs about information requirements, and informs about privacy and confidentiality concerns.
2. Dashboard purpose and concept comprise specification of the purpose of the dashboard, including the context, target user group, and the expected objectives of users of the dashboard.
3. User Interaction and flow specify the intended process of user interaction with the dashboard interface, including the expected user *flow* through the dashboard structure, to gain insight.
4. Data selection and visualization design cover the design and construction of the data visualization, including how the key attributes are selected, their location within the dashboard, and their extraction from the underlying database.
5. Framework architecture covers technical aspects of dashboard framework, including input data structures and interfaces.

In the following section, the execution and design considerations involved in the development of a dashboard framework for HDSS sites are described.

#### Study Setting

The setting of this study is the Africa Health Research Institute (AHRI) in KwaZulu-Natal, South Africa (SA). AHRI’s field site operates as an HDSS, generating high-quality longitudinal datasets to capture the demographic and health changes brought about by the HIV epidemic and evaluate interventions to mitigate their impact [6]. The broad objectives of the dashboard trial were to increase access to information for staff and community members to AHRI’s Somkhele field site, providing a real-time picture of data collection operations and insight into the variety of major trials and surveys ongoing within the region. The variety and velocity of data collection at AHRI means that this setting was similar to other HDSS contexts. In this context, the dashboard framework was implemented for a monitoring Treatment as Prevention (TasP) trial for sociodemographic analysis and intervention cascade progression. In this study, the primary focus will be upon the TasP implementation.

Within this setting, the dashboards would be displayed on 3 large touch screens within the research center. Placing of the dashboards in public areas hoped to encourage collaborative use, visibility, and discussion of datasets, and it could provide a means to explain the work of the institute field site to visitors.

#### Dashboard Purpose and Concept

The purpose of the AHRI data dashboard was to provide users with the opportunity to access information about studies at the site and provide a building block for the development of a framework, increasing the visibility and access to the datasets produced at the sites. The dashboard would give a clear and concise overview of the information collected in an understandable and accessible format while ensuring the anonymity of the participants. A diverse range of users would use the dashboards, yet a dashboard cannot be created to fit every user persona. The aim was to design a dashboard, which anyone working at a site could feel comfortable using to explore core datasets in the context of a specific study, for example, community members, field workers, medical staff and resident scientists, and visiting researchers. This design would enable users to either review overall progress or to drill down into analysis depending on their needs.

In achieving these objectives, the dashboard interface would be required to meet the following design criteria:

- Provide a monitor of key performance indicators (KPIs)
- Enable spatial interrogation of datasets in relation to KPIs to identify trends
- Allow for switching from a global to local view in relation to these indicators
- Drill down to the local regions of the map to explore datasets in greater detail

On the basis of these needs, we developed a 2-stage dashboard comprising an overview page and analysis page. The overview page would provide a global picture of the study in question and would feature a display of the key indicators relating to the progress of the trial and an interactive map to display these indicators to allow rapid identification of trends. Interaction with the overview page would enable users to filter data by indicator and act as a jumping-off point to the analysis page. The analysis page would focus on a selected subdivision within the study site, allowing users to compare the performance of a region to the global area and augment their analysis with the typical datasets produced at an HDSS.

#### User Interaction and Flow

The global performance of indicators is the starting point for the users’ interaction with a dashboard, a set of anchors to guide their exploration of the datasets displayed and to compare performance at the global and local level. The design of the dashboard was based on Shneiderman’s [26] principles of visual information seeking mantra *overview first, zoom and filter, and details on demand*. The overview component of the dashboard is the display of the global indicators combined with the spatial overview provided by the map. The user can filter data by changing the map display by indicator. The map provides a means to zoom to areas of interest on the basis of spatial patterns of variance. Selecting a map region allows the user to access the details for that specific region.

#### Data Selection and Visualization Design

A successful dashboard provides an overall picture of the datasets represented with a key objective in mind. In this context, there are a variety of indicators that can reflect the health of a population, for example, child mortality, life expectancy [10]. For the TasP dashboard, for example, the indicators chosen were aligned with the United Nations Programme on HIV/AIDS 90/90/90 targets across the study region (described in Table 1).

**Figure 1:**
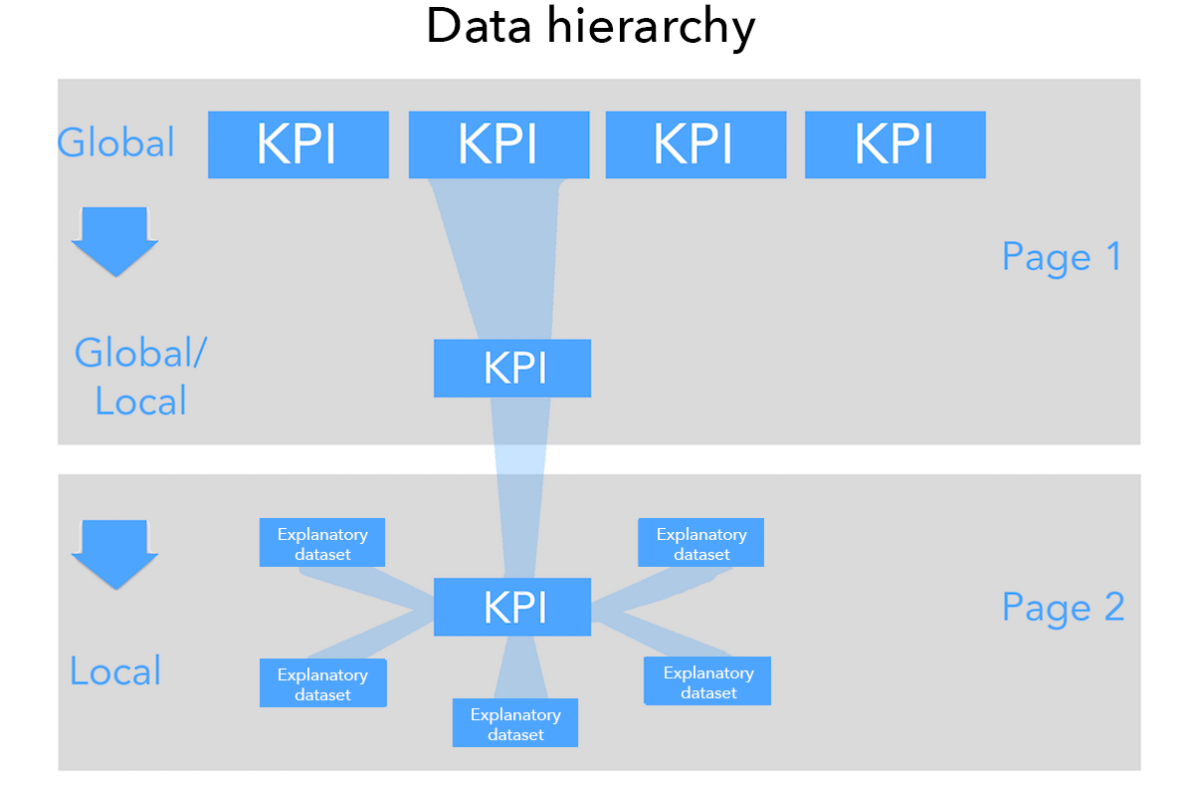
User interaction flow between global and local. This image shows the interaction of key performance indicators (KPIs) at the global and local scales, allowing the user to explore the broad spatial trends in the indicator at the global scale, before drilling down into the finer scale spatial variation and associations at the local.

Framing data exploration through indicators allows the user to keep the global in mind while exploring details. The dashboard’s exploratory hierarchy of datasets is the global view of KPIs than viewing the KPIs at a local level. This flow allows the user to view the global and the local together and make comparisons between both scales, gaining insight into relative local performance and identifying emerging trends quickly and clearly (see Figure 1). Users could directly compare the local performance of a map region with all global indicators and interrogate factors that could influence the rate of the KPIs with the help of explanatory datasets such as demographic data. The user can display explanatory datasets normalized by the region's total population or normalized by population relating to the indicator (eg, HIV-positive individuals).

##### The Map Interface

The overview page was designed to fulfill the function of a traditional dashboard by providing the user with access to the most salient information relating to the subject matter while encouraging a detailed exploration of the datasets. The design intention was to provide the user with an overview of the progress of the study to the KPIs, to review a spatial representation of indicator performance, and provide the means to compare the global performance with the local. Once a region had been selected, the user could move to the analysis page, which would focus on local performance with the aid of explanatory variables and the use of comparative visualizations.

**Table 1:** Summary of selected key performance indicators and data types.

Indicator	Type	Description
Trial participants	Number	The number of individuals enrolled in the Treatment as Prevention trial
HIV prevalence rate	Ratio	The percentage of individuals known to be HIV positive
HIV-positive individuals who know their status/individuals known to be positive	Ratio	The percentage of HIV-positive individuals who know their status/the number of individuals known to be positive
HIV-positive individuals on antiretroviral treatment ART^a^/diagnosed individuals	Ratio	The percentage of HIV-positive individuals on ART
Individuals who are virally suppressed/individuals on ART	Ratio	The percentage of individuals who are virally suppressed

^a^ART: antiretroviral treatment.

Following Shneiderman’s visual information seeking mantra [26], the map page presents an overview of the datasets with the ability to filter according to the KPIs and access local region performance details by using these features to guide the user to the analysis page. The dashboard updates daily from the trial databases, allowing the user to explore the shifting patterns of the study’s progress.

**Figure 2:**
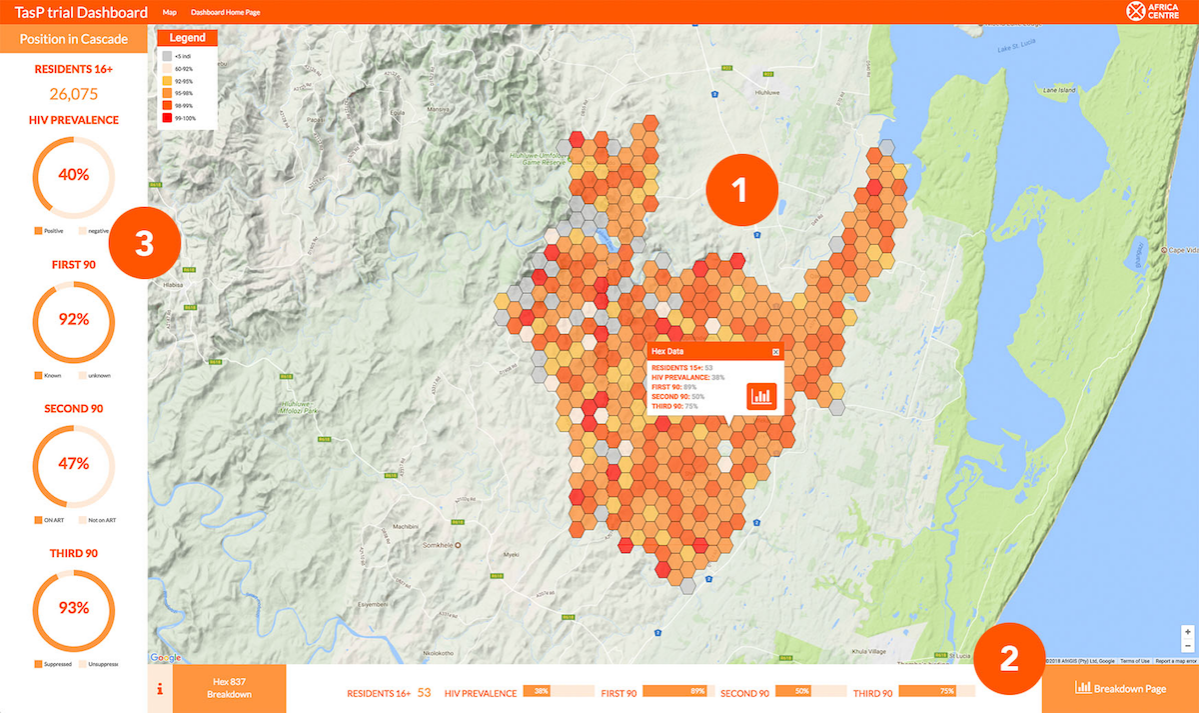
Landing page of the dashboard. This image shows the interactive front page of the dashboard, displaying the global view of 1 indicator within the map (marked 1), measures of key performance indicators (KPIs) at the selected local region at the bottom of the screen (marked 2), and global KPI measures on the left (marked 3). Data are updated in real-time. The user is invited to manipulate the map and change selected regions through click or touch. The Breakdown Page button leads to a more detailed exploration of KPI measures and related factors within the Analysis page.

The overview pages comprise 3 areas of information display (see Figure 2): (1) map, (2) global performance of indicators, and (3) local performance of indicators in a selected region.

The focus of the overview page was the map display (indicated with point 1 in Figure 2). The map provided a spatial view of the study performance through graduated symbols and acted as a vector for the exploration of local regions. Furthermore, one of the weaknesses in the trial’s oversight protocol was the lack of a method to observe spatial variation in the progress of the trial without the generation of geographic information system maps, a time-consuming process. The map would be at the center of the dashboard interface and would drive exploration of trial datasets. The map allows a simultaneous overview of global trends and local variation in the observed attribute. The interface also allows the user to view trial results in relation to local infrastructure, for example, roads and clinics. Spatial data are displayed using a binning technique that divides the trial area into a grid of uniform hexagons. Binning is a means of converting point data into a regular grid of polygons. Each element of the grid represents the aggregation of the points that fall within it. The result is a uniform representation of the trial region, and it allows simple, clear visualization of the datasets, aided by quantification of the map using color breaks. The most pressing concern was to ensure the privacy of the trial’s participants. Privacy was the primary driver for choosing a binning technique as participants were aggregated to a hex, resulting in the highest granularity level of the data being the hex, with all local data displayed in relation to the performance of the hexagon region rather than the individual. The use of a grid acted as a means of distortion that prevented users from directly identifying individual homesteads. The use of a universal grid representing the HDSS region allows for the easy visualization of spatial data within the dashboard framework. Spatial data are aggregated to the hex, and hexes containing no data are not displayed, allowing for comparison across dashboards and providing a method that could be replicated at different sites. A downside of this method is that the grid sections display geographically equal areas rather the population density (eg, skewed toward urbanized areas). A solution was to add a layer that represents population density. Using methods such as size distortion would require the grid to be redrawn for each dashboard, which was not deemed feasible.

The graduated color map is used to identify areas of interest and detect spatial autocorrelation within the trial region. The use of a binning technique also related to practical concerns to create regions to capture local performance and acted as a point of interaction to trigger events on the dashboard. When a hexagon is selected, information relating to indicator performance and the number of trial participants residing in that region is displayed. A button on the information window allows the user to view this region in greater detail on the analysis page. Interacting with a map hexagon also triggers the display of KPIs for the selected region in the page footer. The global performance of indicators is displayed in the left-hand sidebar (point 3 in Figure 2). The sidebar displayed the KPIs in the form of donut charts with the percentage figure contained inside. When an indicator chart interacted with the map display, it would update relating to that indicator, whereas the bottom bar would update to display supplementary information on the selected indicator. Donut charts were selected instead of bar charts, as the visualization allowed for the large display of the percentage behind the chart as trial staff requested the prominent display of targets.

**Figure 3:**
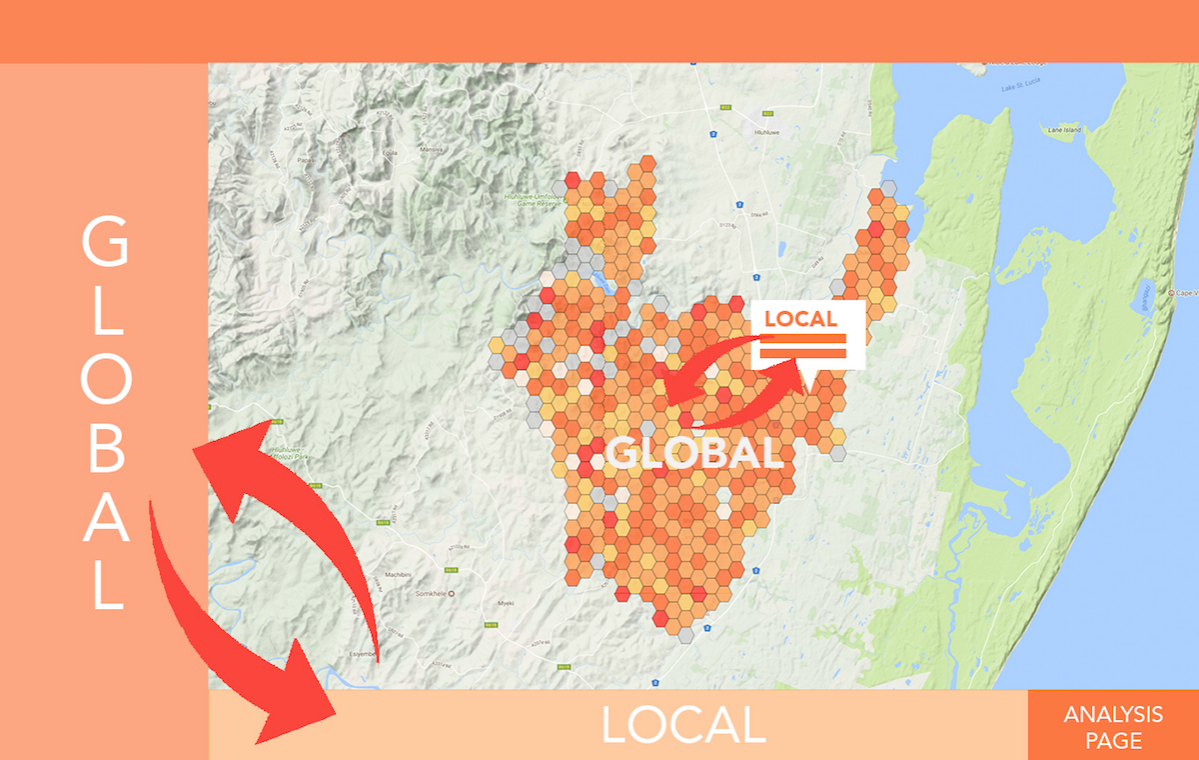
Points of comparison. This image highlights the points of interaction between global and local indicators, showing the points where a user is able to directly compare local and global measures of key performance indicators.

The page footer (point 3 in Figure 2) allows direct comparison between the local indicators and the global, in the left sidebar (see Figure 3). This design was to allow the user to consistently frame the global and local together without overloading them with information. Indicators were displayed using horizontal bar charts and contained an information button to display how the selected map indicator was calculated, and also contained a button that moved the user to the analysis page to explore the KPIs in greater detail with the aid of explanatory datasets.

##### Analysis Page

The analysis page provides a detailed view of the performance of indicators at a local scale, the *details on demand* of Sneiderman’s mantra [26]. The design intention was to view explanatory datasets in relation to the total population resident in each region and to add or remove population data relating to the KPIs. The analysis page allows users to view data relating to HIV status, gender, age group, education, relationship, and economic status. The analysis page facilitates exploration of datasets by allowing the user to explore the demographic differences of the trial participants relating to each indicator. For example, users could compare the education level of HIV-positive individuals who have linked to care with those who have not linked to care. The analysis page allows for in-depth exploration and discussion of the datasets among users.

**Figure 4:**
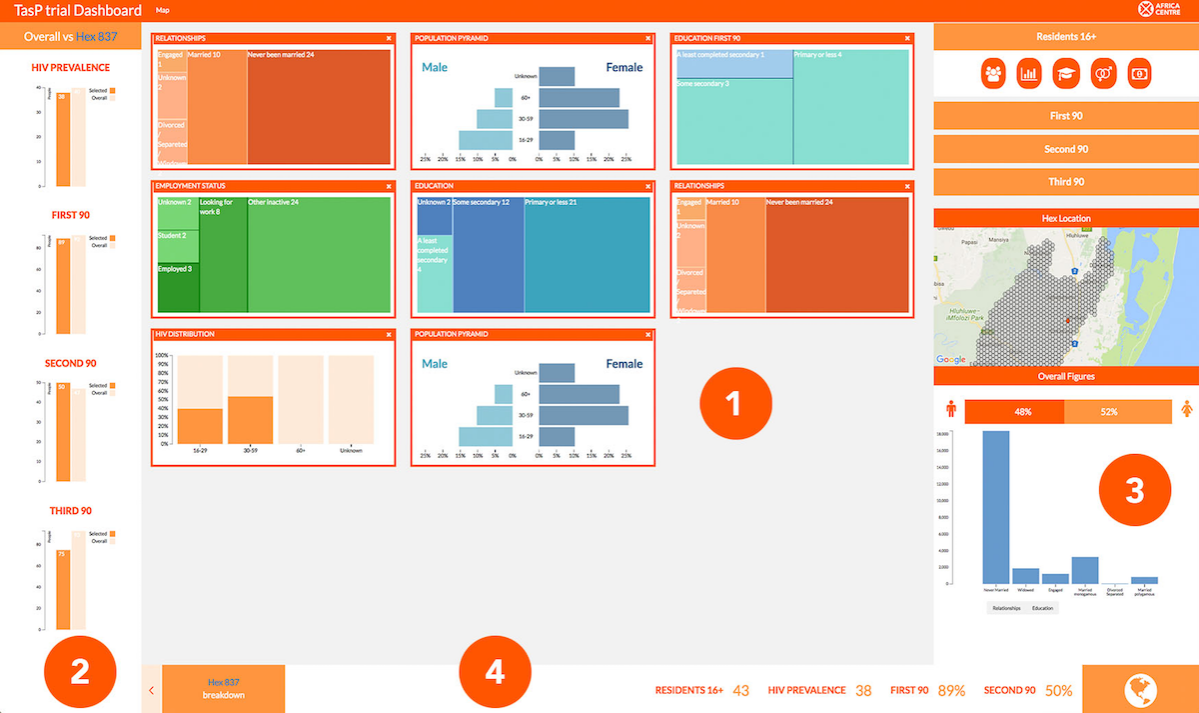
Analysis page. This figure shows the analysis page for a local region selected through the global map page. The page introduces a range of potential explanatory attributes that can be measured against key performance indicators to help develop hypotheses for future studies. Users are able to add and remove visualizations through interactive functionality.

The analysis page comprised 4 areas of information (Figure 4):

1. Explanatory variables
2. Global and local comparison
3. Chart selection, mini map, and global information display
4. Key numbers relating to a region

The focus of the analysis page is the explanatory variables section (point 1 in Figure 4). Within this window, charts are displayed in modules that can be added or removed through interaction with the interface. The user can use a set of charts displaying explanatory datasets relating to all trial participants in the local region. The charts are as follows:

1. A population pyramid
2. A normalized stacked bar chart displaying HIV rates by age group
3. A treemap displaying education levels
4. A treemap displaying relationship status
5. A treemap displaying employment levels

Treemaps were chosen over bar charts as users preferred them, and they allowed for a more precise display of labels relating to data. The user can add the charts display population data in relation to each indicator to explore the differences among participants at different stages of the HIV treatment cascade, allowing the user to compare different outcomes directly. We used multiple visualization methods to exploit their differing strengths to enhance the users’ data exploration and engagement. Additional chart types alongside those listed above were pie, bar charts, and scatter plots.

The left-hand sidebar (point 2 in Figure 4) displays the KPIs in the local region to the global using bar charts, as with the design of the map page allowing the users to keep global and local in their mind while exploring. The right-hand sidebar (point 3 in Figure 4) acts as the control panel for the visualization portal. The user can select charts to add to the center display relating to an indicator. Icons related to the dataset represent chart choices. The sidebar includes a mini-map, highlighting the selected region and a bar chart displaying the global proportional relationships of education, relationship status, and economic status within the trial. This bar at the bottom of the page (point 4 in Figure 4) displays key figures relating to a region, such as a gender breakdown, HIV prevalence, and KPIs.

#### Framework Architecture

The data dashboard was designed to inform the development of a framework. The framework was designed as a modular system to allow for the creation of dashboards by the research data management team at the site. The framework aimed to allow the development of dashboards as new datasets are produced. The aim was to develop new chart options as needed. This modularization comprised 3 components and the retention of these components for each new study.

1. Standard inputs for the dashboard frontend
2. Standard architecture for the inclusion of new datasets into the dashboard
3. Standard dataset formats for the generation of data visualizations

All dashboards run from a Web server within AHRI’s database architecture. Each dashboard is contained within a folder on the Web server, and adding a new folder to the dashboard Web server generates a new dashboard. The dashboards are displayed through a Web browser, and dashboards can be accessed through the dashboard homepage files on the Web server and can be edited by accessing the dashboard folder on the shared drive. The dashboard works as a typical website, and each folder contains index.html, analysis.html, and folders for Cascading Style Sheets (CSS), JavaScript (JS), and datasets. Data managers have access to the folders to edit the dashboard setup for new iterations and troubleshoot errors when they occur. The research data management team was trained on the system through training workshops.

### Ethics Approval and Consent to Participate

Ethics approval was granted for this study by the University of KwaZulu-Natal Biomedical Research Ethics Committee, reference BE497/16. Informed consent to participate in the study was obtained from all participants.

## Results

### Evaluation Design

An evaluation of the dashboard framework design was undertaken using the TasP trial dashboard implementation. This data dashboard was evaluated by assessing its usability with 20 users. The site had 100 workers, and the aim was to engage a broad range of potential users. Participants were selected from people working at AHRI, representative of different groups who would use dashboards to gain insight from AHRI datasets. These groups chosen were as follows.

- Scientific staff: primarily medics, health care specialists, and researchers. Staff that would use dashboards to explore data from a research perspective, easing their access to data.
- Operational staff: those ensuring continued operation of AHRI, focusing on data collection and quality. Staff that would use the dashboards to monitor the progress of data collection in studies.
- Nursing staff: those involved in providing health services in the area. Nurses would use the dashboard to monitor how the data they collect are used and keep up to date with the studies they were involved.
- Community Advisory Board (CAB) members: The CAB comprises members of the local community who are consulted and who advise researchers on the implementations of studies. The dashboards allow the CAB to explore the data collected about the community, supporting the group role in improving public understanding and maintaining accountability.

In total, 5 members of each group were randomly selected.

To assess the usability of the dashboards, the users performed a benchmark task evaluation comprising 5 tasks (each incorporating 2 subtasks) of increasing difficulty, requiring the user to extract information using the dashboards. During benchmark task evaluations, participants use visualizations to perform tasks to extract information, measuring targeted metrics [27,28]. The time taken and accuracy of the user responses for the tasks were recorded to evaluate the dashboard’s usability. The use of a task evaluation can identify possible shortcomings in the representation of data within a visualization system [29]. The task completion component of the evaluation was filmed with the permission of the participants to ascertain the timing of task completion. The tasks undertaken by participants are provided in Multimedia Appendix 1.

Alongside benchmarking, users were also asked to self-evaluate their knowledge of the TasP trial and their level of computer literacy. Participants were also asked to complete a questionnaire using a Likert scale to assess their attitude toward the dashboard, covering the design of the interface, information presentation, and attitudes toward the future use of dashboards within AHRI. The questionnaire is provided in Multimedia Appendix 2.

### Evaluation Results

The results of the evaluation can be broken down into 3 parts: self-evaluation, task evaluation, and the questionnaire.

#### Self-Evaluation

The self-evaluation asked the users to give their level of computer literacy, awareness of the TasP trial, and the dashboard project (see Table 2). Overall, the CAB and nursing staff reported lower levels of computer literacy than the operational and scientific staff, although knowledge of the TasP trial and the dashboard projects varied within groups, with the nursing staff reporting the highest knowledge of the trial, which was expected as the nurses were involved in the clinical component of the TasP trial.

#### Task Completion

**Figure 5:**
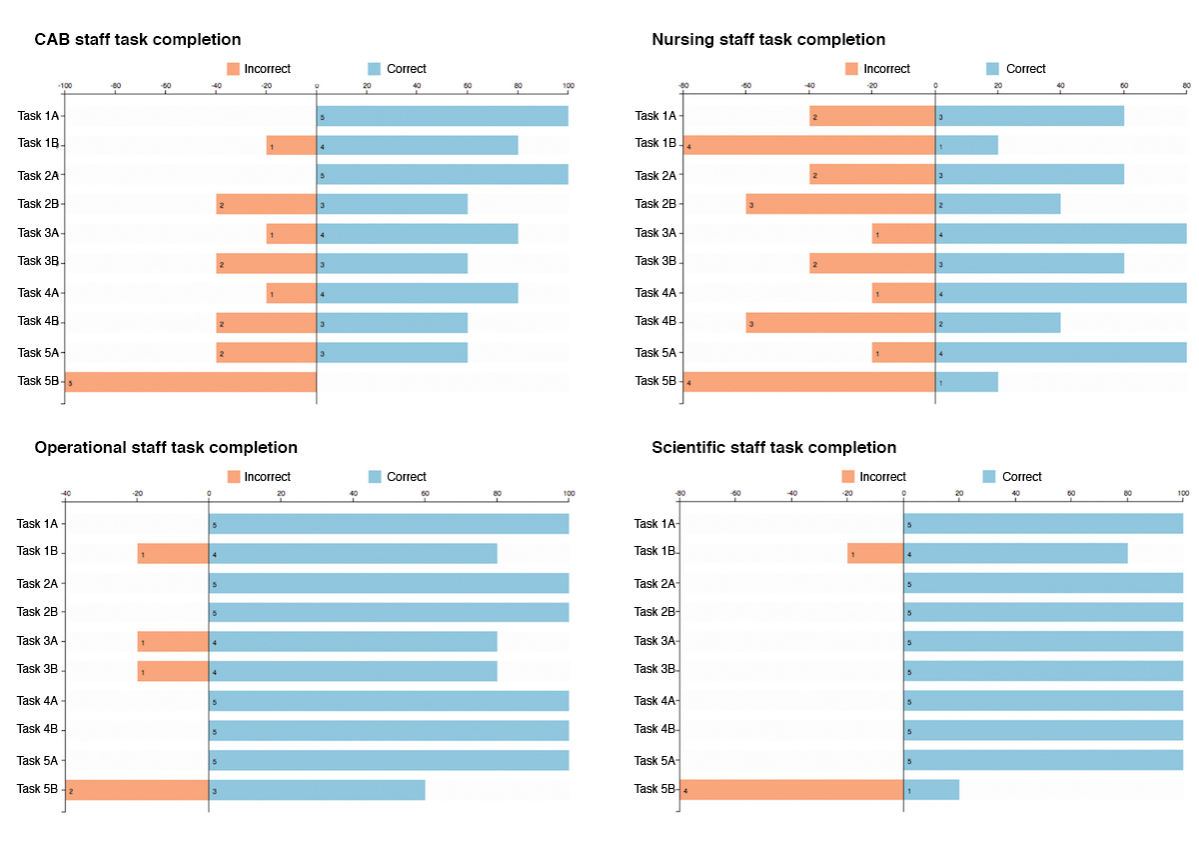
Task completion rates, by group. This figure shows how different groups of participants performed during the evaluation study. CAB: Community Advisory Board.

The results of the task component of the evaluation are provided in Figure 5. The results of the task completion were mostly positive. Except for the second part of the final task, a majority of participants were able to complete the tasks successfully; in the majority of tasks, there was a greater than 70% (14/20) correct completion rate.

The issue with the final task, which involved comparing data from multiple sources, may highlight a design weakness where data must be compared across separate charts rather than as different series within a single chart. Within these results, there was a great deal of variation within the subgroup performance, with the scientific and operational staff performing better in task completion than the CAB and nursing groups. Figure 5 outlines the differences between participant groups.

**Figure 6:**
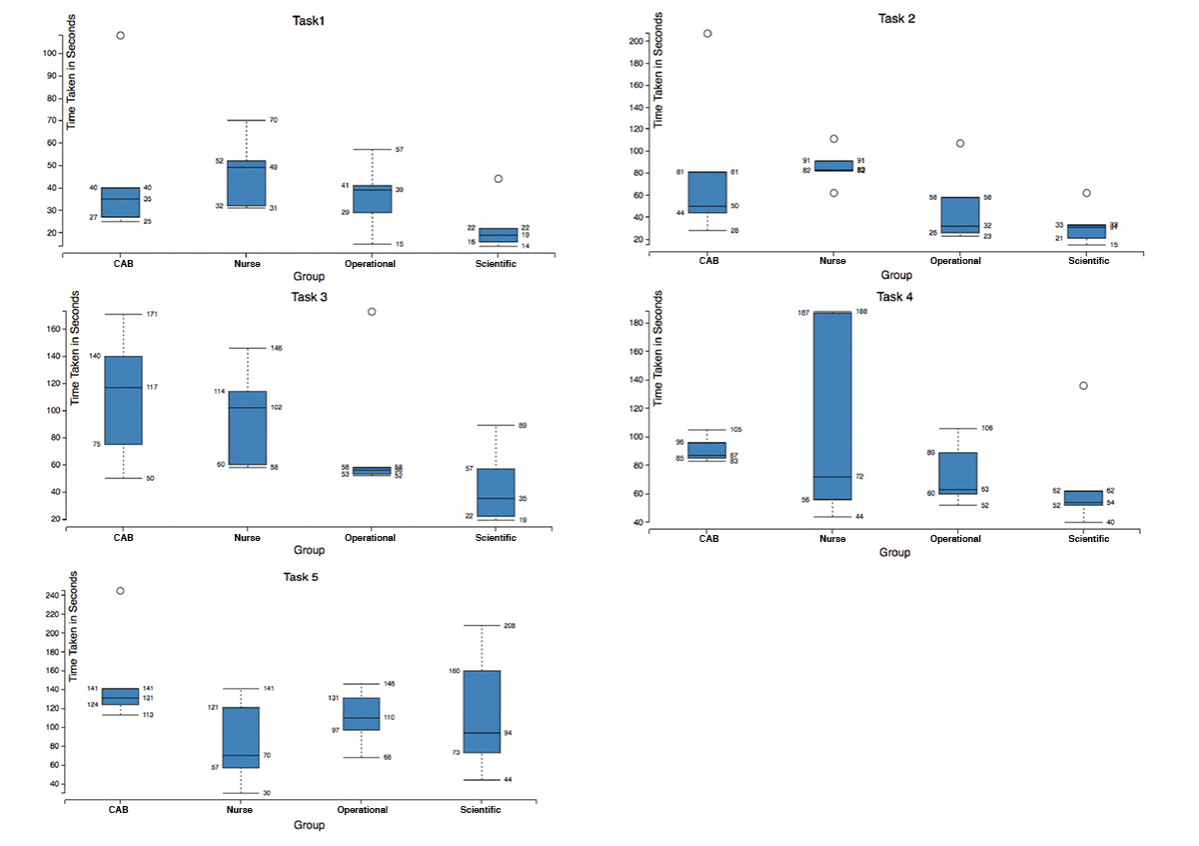
Variance in time taken to complete tasks, by group. This image provides detail on the time taken by each group to complete tasks during the evaluation study. As can be seen, the Community Advisory Board and nursing groups generally took longer to complete tasks than operational and scientific staff, indicating that more work is required around ensuring the dashboard is accessible to all user groups.

Overall the time taken to complete tasks increased with the increasing complexity of each task. On an average, users spent more time on tasks where their answer was incorrect. The scientific and operational staff were generally quicker to answer questions, and more often correct, than the CAB and nursing staff. Through one-way analysis of variance, a significant difference in time taken to complete tasks was found between scientific and nursing staff but not among other groups (Figure 6). These results may be influenced by lower computer literacy on the part of the nursing and CAB participants as self-reported. Therefore, they indicate that there are further steps to go to maximize accessibility across all interested groups.

#### User Questionnaire

The questionnaire comprised 11 questions divided to cover 4 topic areas, the insight provided by the dashboard, the dashboard interface, the future use of the dashboards at the AHRI, and questions allowing users to comment on the dashboards and how they could be improved to provide greater clarity of information. The first 8 questions employed a Likert scale, the last 3 allowed participants to comment on their experience of the dashboard and their thoughts on their future use.

**Table 2:** Responses to self-evaluation of computer literacy from different participant groups.

Participant group		Very low, n	Low, n	Medium, n	High, n	Very high, n
**Community advisory board**						
	Level of computer literacy	0	2	3	0	0
	Knowledge of the TasP^a^ trial	0	3	2	0	0
	Awareness of the dashboard project	0	1	3	0	1
**Nursing staff**						
	Level of computer literacy	0	2	3	0	0
	Knowledge of the TasP trial	0	0	1	4	0
	Awareness of the dashboard project	0	2	2	1	0
**Operational staff**						
	Level of computer literacy	0	0	2	2	1
	Knowledge of the TasP trial	0	1	2	2	0
	Awareness of the dashboard project	1	4	0	1	0
**Scientific staff**						
	Level of computer literacy	0	0	0	5	0
	Knowledge of the TasP trial	0	0	3	2	0
	Awareness of the dashboard project	0	4	1	0	0

^a^TasP: Treatment as Prevention.

Of the users, 80% (16/20) felt that dashboards provided them with a detailed understanding of HIV prevalence and treatment in the TasP trial region, with 5 participants strongly agreeing with the statement and 11 agreeing with the statement. In relation to the questions about the design of the dashboard here, the results were mostly positive, with the majority (60%, 12/20) of users commenting that the terms used in the dashboard aided in the understanding of the data presented, the dashboard was easy to navigate, and the charts were easy to understand. However, the CAB staff and operational staff had a more positive view of the design of the dashboards than the nursing or scientific staff. These differences were also seen in the comments made relating to design and use of dashboards. A member of the nursing staff commented as follows:

It must be used by scientists only, not everyone is familiar with use of graphs and percentages when analysing data.

Nursing staff member

A member of the scientific staff made a similar comment on the presentation of data relating to ability to understand data visualization:

For scientific staff the dashboard is user friendly, for the broader AHRI community, it might not be, particularly for TasP staff, the language and graphs used might not be easy to understand and follow.

Scientific staff member

Through the questionnaire, 95% (19/20) of users agreed that the dashboards were a useful tool for providing information on the studies taking place at the center and that the dashboard should be used more widely as a tool for explaining results of the trials:

Every staff member should be able to use the dashboard for information purposes.

Operational staff member

I think there is enough information in this data dashboard because it is very expansive and could help us to understand easily any information that we need. And would help us to gain more information and if you don’t understand something it is easy for to go to the data dashboard to punch in that information and gain more knowledge.

CAB member

## Discussion

### Principal Findings

In this study, we outlined a framework for the development of a data dashboard for exploiting real-time data within the context of a population health surveillance site, and a mixed-method evaluation demonstrated the frameworks usability. Population health is like many other health disciplines in experiencing a significant increase in the amount of data created at field sites. This increase is driven by new and more efficient data collection systems and techniques, such as mobile and Web platforms and integration of datasets from different sites and studies. The potential of such data could be negated and restricted without a visualization system to disseminate datasets in a manner suitable to a varied set of audiences. These interfaces allow for the rapid detection of emerging health trends, highlighting of outliers, and emerging clusters of activity. Once in place, fewer resources are needed to maintain a real-time monitoring system than is required for the creation of ad hoc analyses. The data dashboard design framework presented here is more widely applicable to other HDSS contexts, where similar challenges are being faced.

The dashboard evaluation demonstrated the potential of the dashboards as a method to explain the ongoing progress of research trials to staff and stakeholders at AHRI. The results of the questionnaire also demonstrated a very positive attitude toward their future use within AHRI across all groups studied, demonstrating that there is enthusiasm for not only the increased visibility of data but also the increased use of data visualization as a means to disseminate datasets.

AHRI is typical of an HDSS site. The success of and enthusiasm for the dashboards speaks to the potential for implementation at other sites. Projects such as the South African Population Research Infrastructure Network, a system of HDSS sites, highlight the importance of data sharing to improve population health outcomes. A standard framework of visualization only aids the cause of sharing and understanding. The results of the evaluation demonstrate the potential for a visualization platform to provide an exploratory interface for users to interrogate multiple data sources, develop insights, and form new research hypotheses within this context. Furthermore, the interface presents an opportunity for collaborations among researchers, through shared data exploration. It also allows stakeholders and community members to see how the data collected in their community are being employed to further research and benefit the community at a large scale. Increasing the visibility of data for community members increases transparency and encourages active participants in ongoing studies. The evaluation demonstrated that although the majority of all groups had positive attitudes toward the increased visibility of data, it is crucial to note variation among groups concerning data needs and user ability.

### Conclusions

The study outlined the design and development of a framework for dashboard design within the context of population health surveillance. The work done thus far can serve as a template for further development. However, further developments in the design and design process are future areas for exploration. The platform could be further developed to allow for increased flexibility in the types of data visualization available and expansion to other sites and contexts. However, it is clear that a more significant step lies in opening up the dashboard to a broader variety of users. Within the current design process, users consulted informally throughout the project, and there would be advantages in exploring user data needs through a user-led process during later iterations. The evaluation demonstrated that users spent more time on a task when they did not complete the task. These results highlight a need to research how different users interact with the same dashboard. The results highlight the need to understand data visualization literacy in the development of platforms for diverse user group and the need to understand the differing information needs of end users. When we talk about dashboards, we often speak about diverse user groups, yet research has shown that the performance of users can differ substantially despite adhering to good design practice. The potential of dashboards to promote the sharing of information and collaboration among diverse users is diminished if we do not consider literacy in design and build user adaptability into future systems.

## Appendix

Multimedia Appendix 1 Evaluation tasks.

Multimedia Appendix 2 User questionnaire.

## Abbreviations

AHRI: Africa Health Research Institute
CAB: Community Advisory Board
HDSS: Health and Demographic Surveillance System
KPI: key performance indicator
SA: South Africa
TasP: Treatment as Prevention
