# Supplementary material for: Developing a Data Dashboard Framework for Population Health Surveillance: Widening Access to Clinical Trial Findings
Source: JMIR Form Res. 2019 Apr 4;3(2):e11342. doi: 10.2196/11342 (PMC6470464; doi:10.2196/11342)
Supplement: Multimedia Appendix 1 [file formative_v3i2e11342_app1.pdf]

TasP Data Dashboard Evaluation

Part 2 - Dashboard Tasks

The part of the evaluation will determine how easy you find it to navigate the dashboard. We will ask you to complete five tasks using the dashboard, entering the responses below. Don't worry if you struggle with any of these questions, this is to test the dashboard not you!

Please enter your responses to each task as indicated below, either by entering text or circling where indicated. If you wish to skip any of the questions, just tick the 'Don't know' option.

Task 1

Identify any region, record the region name and record the number of individuals that know their HIV status.

Region Name
Enter text

Number of Individuals

Don't know

Task 2

Identify any region with a HIV prevalence rate of over 40%. Record the region name, and record this region's performance in the 90/90/90 targets.

Region Name

Region Performance

Don't know

Task 3

Identify any region performing better then the overall trial rate for the second 90. Record the region name, and identify if this region is performing better or worse than overall trial rates in the first 90 and third 90 metrics.

Region Name

Region Performance in First 90 Better or Worse
Please Circle

Region Performance in Third 90 Better or Worse
Please Circle

Don’t know

Task 4

Identify any region performing better then the overall trial rate for the second 90. Record the region name, and drilldown to discover which age group has the highest HIV infection rate.

Region Name

Age Group

Don’t know

Task 5

Identify any region performing better then the overall trial rate for the second 90. Record the region name, and drilldown to record the difference in the education level, if any, the resident 16+ population and the population relating to the second 90 indicator.

Region Name

Difference in Education Level

Don’t know
