# Supplementary material for: Developing a Data Dashboard Framework for Population Health Surveillance: Widening Access to Clinical Trial Findings
Source: JMIR Form Res. 2019 Apr 4;3(2):e11342. doi: 10.2196/11342 (PMC6470464; doi:10.2196/11342)
Supplement: Multimedia Appendix 2 [file formative_v3i2e11342_app2.pdf]

TasP Data Dashboard Evaluation
Part 3 - Usability Questionnaire

In this final part of the evaluation we'll ask you about your feelings about the Dashboard. Again, there are no correct answers here, just tell us what you think!

For each question, circle the option that corresponds to your feelings. If you don't know or are unsure about any response, circle 'Don't know'.

Question 1

Answer how much you agree with the following statement:

The Data Dashboard provided me with a detailed understanding of HIV prevalence and treatment in the TasP trial region.

Strongly Disagree	Disagree	Neither Agree Nor Disagree	Agree	Strongly Agree

Don't know

Question 2

Answer how much you agree with the following statement:

The terms used within the Dashboard were clear, and helped me understand the data.

Strongly Disagree	Disagree	Neither Agree Nor Disagree	Agree	Strongly Agree

Don't know

Question 3

Answer how much you agree with the following statement:

Enough information and hints were provided to help me navigate through the Dashboard.

Strongly Disagree	Disagree	Neither Agree Nor Disagree	Agree	Strongly Agree

Don’t know

Question 4

Answer how much you agree with the following statement:

I found it easy to navigate through the Dashboard

Strongly Disagree	Disagree	Neither Agree Nor Disagree	Agree	Strongly Agree

Don’t know

Question 5

Answer how much you agree with the following statement:

The charts used in the Dashboard were easy to understand.

Strongly Disagree	Disagree	Neither Agree Nor Disagree	Agree	Strongly Agree

Don’t know

Question 6

Answer how much you agree with the following statement:

The design and colour scheme of the Dashboard heightened my interest and desire to use it.

Strongly Disagree	Disagree	Neither Agree Nor Disagree	Agree	Strongly Agree

Don’t know

Question 7

Answer how much you agree with the following statement:

The design of the map did not help me understand variation in indicators across the study region.

Strongly Disagree	Disagree	Neither Agree Nor Disagree	Agree	Strongly Agree

Don’t know

Question 8

Answer how much you agree with the following statement:

Data Dashboards are a useful tool for providing information to scientists.

Strongly Disagree	Disagree	Neither Agree Nor Disagree	Agree	Strongly Agree

Don’t know

Question 9

Answer how much you agree with the following statement:

Data Dashboards should be used more widely across the Africa Centre as a tool for explaining the results of trials.

Strongly Disagree	Disagree	Neither Agree Nor Disagree	Agree	Strongly Agree

Don’t know

Question 10

In the space below, provide any suggestions about further information or data that could be added to help improve your understanding.

Question 11

In the space below, please provide any further comments or opinions on the Dashboard.
